# The mitochondrion as a mechanometabolic conductor of cancer metastasis: pharmacological insights from the mitochondria–peroxisome interface

**DOI:** 10.3389/fphar.2026.1983363

**Published:** 2026-10-27

**Authors:** Subhajit Dutta, Namrata Maity

**Affiliations:** 1 Department of Biochemistry and Molecular Cell Biology (IBMZ), Center for Experimental Medicine, University Medical Center Hamburg-Eppendorf, Hamburg, Germany; 2 Department of Molecular Biology, Ariel University, Ariel, Israel

**Keywords:** cancer metastasis, ferroptosis, mechanometabolism, mitochondria, organelle dynamics, peroxisomes, pharmacological selectivity, redox signalling

## Abstract

Metastasis demands more than sustained ATP production: cancer cells must move organelles, remodel membranes, manage oxidative stress, and reprogram metabolism as they pass from invasion to distant colonisation. Mitochondria sit at the centre of this adaptation. This Review organises their contribution around four linked axes: deployment to the cell cortex and invadopodia; reactive oxygen species and metabolite signalling; VDAC1-dependent metabolite, calcium, apoptotic and mtDNA gating; and HIF-1α–BNIP3-dependent remodelling of the mitochondrial pool. We then ask how this mitochondrial programme is altered by peroxisomes. The two organelles share components of the fission machinery, coordinate lipid-derived substrate use and cellular redox balance, and divide tasks in hydrogen-peroxide control, ether-lipid synthesis, organelle quality control and MAVS signalling. These connections matter pharmacologically. A DRP1- or redox-directed intervention may affect both organelles, whereas VDAC1 provides a more mitochondria-restricted target logic. Peroxisomal pathways are also context dependent: fatty-acid shortening and ether-lipid synthesis can increase metabolic flexibility and membrane remodelling, yet the same pathways may raise oxidative stress or create ferroptotic susceptibility. By separating established molecular links from mechanisms that remain untested in metastatic models, we propose a hierarchical view in which mitochondria remain the principal mechanometabolic conductor, while peroxisomes set important boundary conditions for substrate use, redox signalling, immune responses and therapeutic selectivity. We also state the limitation this framing carries. The mitochondrial arm of the framework rests on direct experimental evidence, whereas a causal contribution of mitochondria–peroxisome contacts to invasion, transit or colonisation has not been demonstrated: it is inferred here from contact biology characterised without testing metastatic endpoints, from division machinery shared between the two organelles, and from biochemical coupling. The interface is therefore advanced as a testable framework for target selection and pharmacological attribution, not as an established driver of metastasis.

## 1 Introduction

Metastasis is the major cause of cancer mortality and reflects the ability of tumour cells to leave the primary site, enter into the bloodstream or lymphatics, and establish colonies in distant organs (Wang et al., 2023). Each step of this cascade imposes distinct bioenergetic and biosynthetic demands: a cell must generate sufficient mechanical force to breach the basement membrane, sustain directional motility through mechanically heterogeneous stroma, survive anoikis in the circulation, and adapt its metabolism to a foreign organ microenvironment. For decades, the Warburg effect—the preferential use of aerobic glycolysis despite available oxygen—was interpreted as evidence that mitochondria were functionally diminished in tumour cells. This view has been revised. Mitochondria in cancer cells are dynamically regulated organelles whose localisation, morphology and output can be tuned to support different stages of metastatic dissemination.

We use the term “mechanometabolic” throughout this review to describe the dual capacity of mitochondria to simultaneously supply the mechanical energy (ATP for actin dynamics, myosin contractility, and membrane remodelling) and the metabolic signals (ROS, oncometabolites, Ca^2+^) that together enable the physical work of invasion and the transcriptional reprogramming required for metastatic competence. This concept captures what distinguishes the mitochondrial contribution from purely metabolic or purely signalling models: the organelle functions as an integrated hub where energy production and signal generation are spatiotemporally coupled at sites of active cell movement.

The evidence for mitochondrial centrality in metastasis is now substantial, although the specific adaptations described below are not universal—they vary across tumour types, genetic contexts, and stages of the metastatic cascade (Awad and Abdul Karim, 2025; Picard and Shirihai, 2022; Wang et al., 2023). Metastatic cells frequently exhibit higher mitochondrial membrane potential (ΔΨm), greater oxidative phosphorylation (OXPHOS) capacity, and more dynamic fission–fusion cycling than their non-metastatic counterparts, although these features have been characterised most thoroughly in breast, melanoma, and hepatocellular carcinoma models (Awad and Abdul Karim, 2025; Wang et al., 2023). Mitochondria physically relocate to sites of high energy demand—lamellipodia, invadopodia, and the leading edge—where local ATP production drives the actin polymerisation and matrix metalloprotease (MMP) secretion required for tissue invasion (Awad and Abdul Karim, 2025; Wang et al., 2023). Simultaneously, mitochondria generate calibrated bursts of reactive oxygen species (ROS) that activate redox-sensitive transcription factors including nuclear factor kappa-light-chain-enhancer of activated B cells (NF-κB), activator protein 1 (AP-1), and hypoxia-inducible factor 1α (HIF-1α), each of which transcriptionally reinforces the invasive phenotype (Liu et al., 2023; Zhuang et al., 2023).

Two axes that have received comparatively limited integrative attention in prior reviews are given particular emphasis here, although their relative importance varies across tumour contexts. First, voltage-dependent anion channel 1 (VDAC1), the most abundant channel of the outer mitochondrial membrane (OMM), provides a major pathway for ions and water-soluble metabolites between the cytosol and the intermembrane space; substrate access to the matrix then depends on inner-membrane carriers and channels. In many tumours, VDAC1 supports mitochondrial–glycolytic coupling through hexokinase II, contributes to calcium transfer at mitochondria–ER contacts and regulates interactions with apoptotic proteins. VDAC1 oligomers have also been implicated in mtDNA-fragment release in selected stress models, but this route must be distinguished from the established BAX/BAK macropore pathway of apoptotic mitochondrial outer-membrane permeabilisation (Huang et al., 2025; McArthur et al., 2018; Shoshan-Barmatz and Mizrachi, 2012; Shoshan-Barmatz et al., 2017a). Second, the hypoxia–HIF-1α axis redirects carbon use and regulates mitochondrial quality through BNIP3- and BNIP3L/NIX-dependent mitophagy (Poole and Macleod, 2021; Vara-Pérez et al., 2021; Zhuang et al., 2023). We emphasise these pathways not as universal master regulators, but as convergence points at which metabolic exchange, organelle quality, apoptosis and inflammatory signalling intersect.

This review integrates mitochondrial spatial deployment, ROS signalling, PGC-1α-mediated biogenesis, organelle remodelling and intercellular mitochondrial transfer into a framework in which the mitochondrion acts as a mechanometabolic conductor of metastatic progression (Figure 1). That framework is not organelle autonomous. Peroxisomes share selected membrane-remodelling proteins with mitochondria, process fatty acids that are inefficient mitochondrial substrates, initiate ether-lipid synthesis and function as both sources and sinks of H_2_O_2_. In mammalian cells, MFN1 and MFN2 can promote mitochondria–peroxisome clustering (Huo et al., 2022), whereas contact-dependent transfer of oxidants from stressed mitochondria to antioxidant-rich peroxisomes has been demonstrated in cell models without testing metastatic endpoints (DiGiovanni et al., 2025). These links place peroxisomes in a position to modify mitochondrial performance and drug response, even though their direct contribution to metastatic behaviour remains less firmly established. The sections that follow therefore preserve a mitochondrial hierarchy while examining the interface wherever it changes mechanistic attribution, therapeutic selectivity or vulnerability.

**FIGURE 1:**
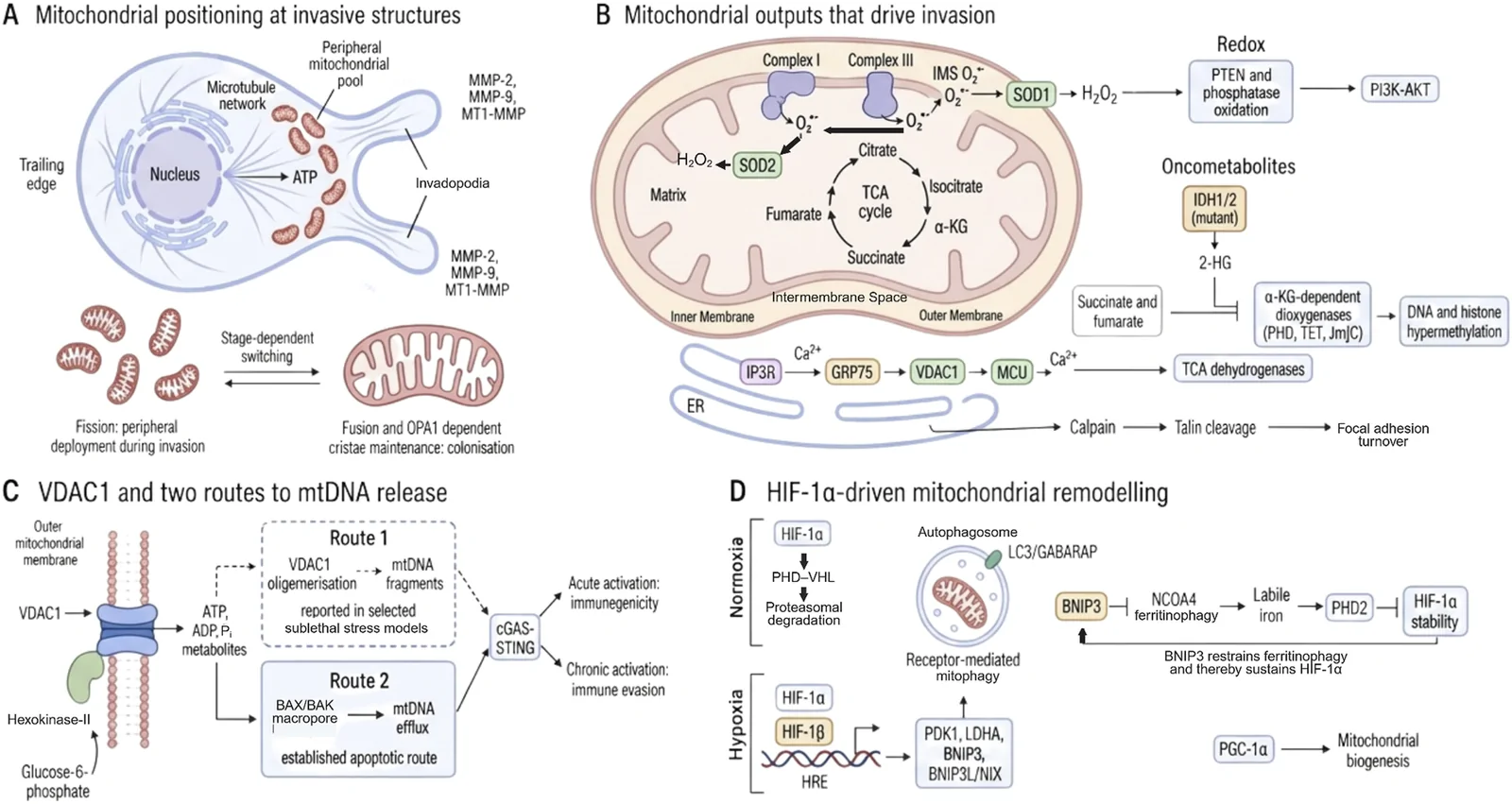
Four mitochondrial axes that support metastatic progression. **(A)** Mitochondrial positioning at invasive structures supports local ATP supply and protease delivery; fission favours peripheral deployment during invasion, whereas OPA1-dependent cristae maintenance supports respiratory competence. **(B)** Complex I releases superoxide into the matrix and Complex III to both sides of the inner membrane; matrix O_2_•^-^ is dismutated by SOD2 and intermembrane-space O_2_•^-^ by SOD1, producing H_2_O_2_ that can oxidise PTEN and protein tyrosine phosphatases upstream of PI3K-AKT. Mutant IDH1/2, succinate and fumarate converge on α-ketoglutarate-dependent dioxygenases; IDH1 is cytosolic and IDH2 mitochondrial, although they are grouped for clarity. ER Ca^2+^ transfer through IP3R-GRP75-VDAC1 and MCU links MERCs to matrix metabolism and focal-adhesion turnover. **(C)** VDAC1 supports outer-membrane metabolite exchange and HK-II coupling. VDAC1 oligomerisation is shown as a selected-stress route to mtDNA fragments, distinct from the established BAX/BAK macropore pathway; both can converge on cGAS-STING. **(D)** Normoxic PHD-VHL signalling promotes HIF-1α degradation; hypoxia stabilises HIF-1α/HIF-1β transcriptional activity, including BNIP3/BNIP3L-NIX-dependent mitophagy. BNIP3 also restrains ferritinophagy and thereby supports HIF-1α stability.

This uneven evidence base creates a practical pharmacological problem. DRP1, MFF and FIS1 are routinely discussed as mitochondrial fission proteins, but they also participate in peroxisome division (Itoyama et al., 2013; Koch and Brocard, 2012; Koch et al., 2005; Schrader et al., 2022). Whole-cell ROS measurements cannot separate ETC-derived oxidants from an ACOX1-dependent peroxisomal contribution, nor from oxidants transferred between the two compartments (Chen et al., 2018; DiGiovanni et al., 2025), and ferroptosis depends partly on ether phospholipids whose synthesis begins in peroxisomes (Benjamin et al., 2013; Stazi et al., 2019; Zou et al., 2020). An apparently mitochondrial drug phenotype may therefore reflect dual-organelle activity or a non-organelle off-target.

The selectivity problem can be stated precisely at the outset, because it is the argument this Review is built around. DRP1 is recruited to mitochondria and to peroxisomes through the shared adaptors FIS1 and MFF, whereas MIEF1 and MIEF2 (MiD51 and MiD49) are not targeted to the peroxisomal surface and act only at mitochondria (Itoyama et al., 2013; Koch and Brocard, 2012; Koch et al., 2005; Palmer et al., 2013; Schrader et al., 2022). The widely used inhibitor P110 blocks the DRP1–FIS1 interaction while sparing DRP1’s other adaptors (Qi et al., 2013). P110 is therefore adaptor-selective but organelle-agnostic, because the adaptor it selects is the shared one, and MIEF1/2 rather than FIS1 is the genuinely mitochondria-selective node. The same asymmetry recurs for redox enzymes, ether-lipid synthesis and selective autophagy. This single comparison is what converts the mitochondria–peroxisome interface from a descriptive network into a target-selection problem; it is developed, with the experiments that would test it, in Section 11.5.1, and the intervening sections establish the mitochondrial biology on which it depends.

The Review remains deliberately mitochondrial in hierarchy. Spatial ATP supply, VDAC1-mediated gating, mitochondrial calcium handling, HIF-1α-dependent remodelling and intercellular mitochondrial transfer form the primary framework. Peroxisomes are brought into the discussion where they alter the interpretation of those mechanisms, expose a selectivity constraint, or create a complementary vulnerability, and are treated as a genuine control layer rather than an appended catalogue of peroxisomal functions.

## 2 Spatial deployment of mitochondria at invasive structures

### 2.1 Mitochondrial trafficking to the leading edge

Mitochondria redistribute towards the leading edge and other cortical regions of high energy demand during migration. AMPK can couple local ATP consumption to recruitment, while MIRO/TRAK/kinesin machinery controls long-range transport (Cunniff et al., 2016; Schuler et al., 2017). Restricting mitochondrial trafficking lowers local ATP availability, focal-adhesion turnover and invasion, although glycolysis can also supply cortical ATP; the relative contribution therefore depends on tumour lineage and nutrient state rather than reflecting an absolute requirement for oxidative phosphorylation.

### 2.2 Invadopodia biogenesis and mitochondrial ATP supply

Invadopodia are actin-rich protrusions that concentrate MMP-2, MMP-9 and MT1-MMP for focal extracellular-matrix degradation. Mitochondria accumulate near invasive protrusions, and restricting their trafficking reduces matrix invasion (Caino et al., 2016; Cunniff et al., 2016; Schuler et al., 2017). Local ATP supports actin turnover and nucleotide-dependent protease trafficking. N-WASP is a nucleation-promoting factor rather than an ATPase; ATP binding contributes to Arp2/3 activation, whereas ATP hydrolysis occurs after branch formation and promotes branch ageing and dissociation (Martin et al., 2006). Thus mitochondrial ATP can support several coupled invasive processes without implying that glycolytic ATP is generally insufficient.

### 2.3 Mitochondrial dynamics and stage-dependent migratory competence

Mitochondrial morphology is coupled to migration in a stage- and context-dependent manner. ERK1/2-dependent DRP1 phosphorylation promotes fragmentation, peripheral trafficking and invasion in several RAS/MAPK-driven models, while a soft matrix can drive DRP1- and MIEF1/2-dependent fission, mitochondrial ROS and NRF2-mediated chemoresistance (Kashatus et al., 2015; Romani et al., 2022; Serasinghe et al., 2015). Fission can facilitate deployment of smaller mitochondrial units, whereas OPA1-dependent cristae maintenance can sustain respiration and metastatic-cell fitness (Diokmetzidou et al., 2025). The relevant trait is therefore plasticity between fission-dominant migration and fusion/cristae-supported survival or colonisation. Because DRP1, MFF and FIS1 also participate in peroxisome division, organelle attribution requires paired measurements (Sections 6.2 and 11.5.1).

## 3 Mitochondrial outputs that drive invasion

### 3.1 Reactive oxygen species as pro-invasive second messengers

Mitochondrial ROS have a dose-, species- and compartment-dependent relationship with metastasis. Moderate oxidant signals can reinforce invasion, whereas excessive ROS causes damage or death; antioxidant reserve helps define this functional window (Liu et al., 2023; Wang et al., 2023).

Complexes I and III generate superoxide that is dismutated by SOD2 in the matrix and SOD1 in the intermembrane space. H_2_O_2_ can then reach cytosolic targets, where oxidation of PTEN or protein tyrosine phosphatases prolongs PI3K-AKT and receptor signalling (Murphy, 2009). Local cofilin oxidation inhibits severing and can stabilise leading-edge actin, rather than simply ‘activating’ cofilin (Cameron et al., 2015). Mitochondrial ROS can also support NF-κB and HIF-1α signalling; because the proximal chemistry is context dependent, we describe these pathways at the level established experimentally rather than assigning a universal oxidation event (Chandel et al., 2000; Liu Y. et al., 2020a; Weinberg et al., 2010).

### 3.2 Metabolite signalling: succinate, fumarate, and oncometabolites

TCA-cycle metabolites become signals when altered enzyme activity or flux causes accumulation. Succinate in SDH-deficient tumours inhibits α-ketoglutarate-dependent dioxygenases, while fumarate after FH loss additionally succinates KEAP1 and activates NFE2L2/NRF2-dependent stress programmes (Adam et al., 2011; Xiao et al., 2012). Mutant IDH1/2 produces 2-hydroxyglutarate, which inhibits TET and JmjC demethylases and other α-ketoglutarate-dependent enzymes, promoting DNA and histone hypermethylation. These mechanisms are strongest in tumours carrying the corresponding metabolic lesions and should not be generalised to all cancers. Mitochondrial-derived peptides provide an additional signalling layer; exogenous MOTS-c suppresses ovarian-cancer migration and invasion in reported models (Yin et al., 2024).

### 3.3 Mitochondrial calcium and invasion

Ca^2+^ transfer at mitochondria-ER contact sites links metabolism to motility. IP3R releases ER Ca^2+^, GRP75 bridges IP3R to VDAC1, VDAC1 conducts Ca^2+^ into the intermembrane space and MCU carries it into the matrix, where Ca^2+^-sensitive TCA dehydrogenases increase NADH production and respiratory flux (Baughman et al., 2011; Shoshan-Barmatz and Ben-Hail, 2012; Shoshan-Barmatz et al., 2017a). Cytosolic Ca^2+^ also activates calpain-dependent talin cleavage during focal-adhesion turnover (Franco et al., 2004). MCU is upregulated in colorectal cancer and can support growth through TFAM-dependent mitochondrial biogenesis as well as acute metabolic effects (Liu Y. et al., 2020a). The IP3R-GRP75-VDAC1 complex is discussed in Section 4.3.

### 3.4 Peroxisomal modulation of the mitochondrial ROS window

The mitochondrial ‘ROS window’ becomes more complex when peroxisomes are included. ACOX enzymes generate H_2_O_2_ during peroxisomal β-oxidation, whereas catalase removes it; peroxisomes can therefore act as oxidant sources or sinks depending on flux and antioxidant reserve. SIRT5 desuccinylates ACOX1 and restrains its activity (Chen et al., 2018). The frequently quoted estimate that peroxisomes account for roughly one-third of cellular H_2_O_2_ derives from reconstructed rat-liver subcellular fractions rather than intact cells and is tissue-specific (Boveris et al., 1972); Figure 2 therefore does not present a universal numerical fraction.

**FIGURE 2:**
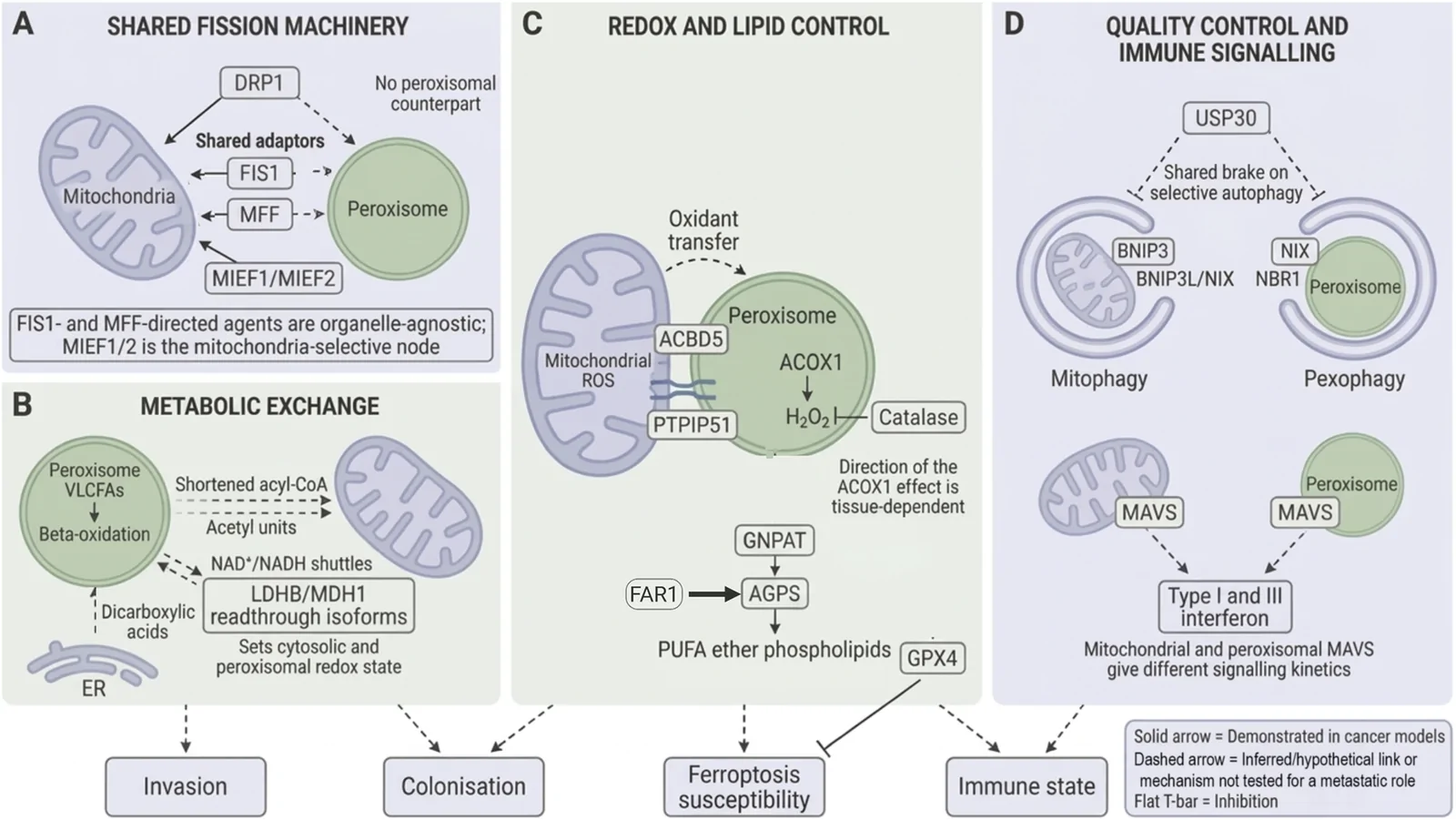
The mitochondria-peroxisome interface as a regulatory layer of mitochondrial mechanometabolism. **(A)** DRP1 is recruited to both organelles through shared FIS1/MFF, whereas MIEF1/MIEF2 (MiD51/MiD49) are mitochondria-specific. **(B)** Peroxisomal β-oxidation shortens fatty acids and exchanges acyl/acetyl units and NAD(H)-linked reducing equivalents with the wider cell. **(C)** ACBD5-PTPIP51-mediated oxidant transfer is shown dashed because its contribution to metastasis has not been demonstrated; ACOX1 produces H_2_O_2_, catalase removes it, and FAR1/GNPAT/AGPS support PUFA ether-phospholipid synthesis. GPX4 inhibits ferroptosis susceptibility rather than ether-lipid synthesis itself. **(D)** Mitophagy, pexophagy and MAVS signalling illustrate shared quality-control and immune outputs. Solid arrows denote interactions demonstrated in cancer models; dashed arrows denote inferred/hypothetical links or mechanisms whose role in metastasis remains untested. Phenotype connectors to invasion, colonisation, ferroptosis susceptibility and immune state are dashed to indicate proposed links within this framework.

Mitochondrial and peroxisomal redox systems also communicate. ACBD5-PTPIP51 contacts can transfer mitochondrial oxidants to peroxisomes for detoxification, although this was demonstrated outside metastatic cancer (DiGiovanni et al., 2025). Cancer data show context dependence: increasing ACOX1-dependent oxidation after SLC9A5 knockdown suppresses colorectal-cancer proliferation and invasion, whereas SIRT5 loss in hepatocellular carcinoma increases ACOX1 succinylation and activity, H_2_O_2_ production and oxidative damage (Chen et al., 2018; Zheng and Wang, 2023). Organelle-targeted sensors and selective manipulation of ETC sites, ACOX1 or catalase are therefore required to assign a migration phenotype to its initiating redox compartment.

## 4 VDAC1: the metabolic gate of the outer mitochondrial membrane

### 4.1 Structure and canonical functions

VDAC1 is a ∼32-kDa β-barrel channel and major aqueous pathway across the outer mitochondrial membrane. ATP, ADP, respiratory substrates, ions and other water-soluble metabolites exchange between cytosol and intermembrane space through VDAC channels, with inner-membrane carriers controlling matrix access (Shoshan-Barmatz and Ben-Hail, 2012; Shoshan-Barmatz and Mizrachi, 2012). VDAC1 also scaffolds proteins including HK-II and BCL-2-family regulators, placing it at the intersection of metabolism and apoptosis; voltage-dependent conductance states observed in reconstituted membranes should not be treated as a simple in-cell switch (Camara et al., 2017; Shoshan-Barmatz et al., 2015).

### 4.2 VDAC1 overexpression in cancer and the Warburg phenotype

VDAC1 is elevated in multiple solid-tumour models and cohorts and can support glycolytic-mitochondrial coupling through HK-II (Arif et al., 2024; Shoshan-Barmatz et al., 2015; 2017b). Matrix-derived ATP reaches mitochondria-bound HK-II through ANT and VDAC1, while the VDAC1-HK-II association can also influence apoptotic sensitivity (Haloi et al., 2021; Krasnov et al., 2013). Peptides that disrupt this interface restore apoptotic sensitivity in preclinical models and provide a more restricted target logic than indiscriminate channel blockade (Liu et al., 2019; Sun et al., 2025; Zhang et al., 2021).

### 4.3 VDAC1 in calcium transfer at MERCs

At MERCs, IP3R releases ER Ca^2+^, GRP75 bridges IP3R to VDAC1, VDAC1 conducts Ca^2+^ into the intermembrane space and MCU transfers it into the matrix. Matrix Ca^2+^ stimulates TCA dehydrogenases and can increase NADH production and oxidative metabolism; disruption of the complex reduces mitochondrial Ca^2+^ uptake and bioenergetic responses (Baughman et al., 2011; Shoshan-Barmatz and Ben-Hail, 2012; Shoshan-Barmatz et al., 2017a). Sustained transfer at high loads can instead trigger permeability transition. Sections 3.3 and 7.1 refer to this account rather than repeating it.

### 4.4 VDAC1 oligomerisation, mitochondrial permeabilisation, and metabolic reprogramming upon silencing

Mitochondrial outer-membrane permeabilisation can proceed through distinct routes. VDAC1 oligomers have been implicated in mtDNA-fragment release in selected sublethal stress models, whereas expanding BAX/BAK macropores provide a well-characterised apoptotic route through which the inner membrane can herniate and expose matrix mtDNA to the cytosol (Huang et al., 2025; McArthur et al., 2018). Their relative contributions in metastatic cancer remain unresolved.

Cytosolic mtDNA can activate cGAS-STING, but outcome depends on signal magnitude, duration and responding cell type: acute activation can enhance tumour immunogenicity, whereas persistent signalling can support inflammatory programmes associated with immune evasion or dissemination. VDAC1 should therefore be treated as one candidate regulator rather than the universal mtDNA-release pore. VDAC1 silencing reduces respiration, proliferation, stemness-associated markers and invasion in several preclinical tumour models, but delivery, isoform compensation and normal-tissue functions constrain translation (Arif et al., 2024; Shoshan-Barmatz et al., 2017b).

## 5 HIF-1α and hypoxia-driven mitochondrial remodelling

### 5.1 The hypoxia response and mitochondrial suppression

Under normoxia, PHD-dependent hydroxylation targets HIF-1α for VHL-mediated ubiquitination and proteasomal degradation. As oxygen falls, PHD activity decreases, HIF-1α accumulates and dimerises with HIF-1β at hypoxia-response elements. Relevant targets include PDK1 and LDHA, which redirect carbon away from mitochondrial oxidation, and BNIP3/BNIP3L-NIX, which promote mitophagy (Poole and Macleod, 2021; Zhuang et al., 2023). Together these responses reduce oxygen demand and remove damaged mitochondria, although their net effect varies with tumour context.

### 5.2 Pseudohypoxia and mitochondrial post-translational adaptation

HIF-1α can also be stabilised without severe hypoxia through oncometabolites, mitochondrial ROS, PI3K-AKT-mTOR signalling, or loss of VHL/PTEN (Zhuang et al., 2023). In metastatic melanoma, mitochondrial ROS sustains HIF-1α under mild hypoxia, while HIF-1α suppresses cyclophilin D through miR-23a/27a, generating a reciprocal circuit that supports metastatic behaviour (Park et al., 2025). This places CypD downstream of a ROS-HIF feedback loop rather than supporting reduced ROS as a mechanism of PHD inhibition.

Mitochondrial retrograde signalling more broadly couples organelle state to nuclear transcription (Picard and Shirihai, 2022). Post-translational control is similarly context dependent: SIRT5 suppresses the Warburg phenotype in renal-cell carcinoma but promotes HIF-1α-dependent adaptation and metastasis in colorectal cancer through PPA2 desuccinylation (Liu et al., 2021; Zhang et al., 2025). Such examples argue against treating any single mitochondrial modifier as uniformly pro- or anti-metastatic.

### 5.3 BNIP3-mediated mitophagy in hypoxic adaptation

BNIP3 is an OMM-localised mitophagy receptor that binds LC3/GABARAP; BNIP3L/NIX is a related HIF-regulated receptor, and dimerisation contributes to receptor activity (Gao et al., 2020; Li et al., 2022; 2026; Marinkovic et al., 2021). By removing depolarised mitochondria, BNIP3/NIX can lower oxidative burden and oxygen consumption. In melanoma, BNIP3 also restrains NCOA4-dependent ferritinophagy: BNIP3 loss increases labile iron, promotes PHD2-dependent HIF-1α degradation and reduces tumour growth, with HIF-1α re-expression rescuing the phenotype (Vara-Pérez et al., 2021). Thus BNIP3 links mitochondrial quality control to hypoxic adaptation and iron homeostasis.

BNIP3/NIX abundance is itself regulated by quality-control pathways, including SCF-FBXL4, while CDK9 inhibition can reduce BNIP3 expression through the SIRT1-FOXO3-BNIP3 axis (Cao et al., 2023; Yao et al., 2022).

### 5.4 PGC-1α and mitochondrial biogenesis: a double-edged sword

PGC-1α coordinates mitochondrial biogenesis and oxidative metabolism through nuclear respiratory programmes, but its metastatic effect is lineage dependent. In breast-cancer models, PGC-1α supports OXPHOS, mitochondrial biogenesis and metastatic colonisation (LeBleu et al., 2014); in melanoma, PGC-1α-high states increase mitochondrial capacity yet suppress invasion (Luo et al., 2016; Vazquez et al., 2013). This contrast is consistent with different demands during invasion, transit and colonisation rather than a universal direction of effect. PGC-1α-driven biogenesis in stromal cells can also increase mitochondria available for transfer to melanoma cells (Kumar et al., 2022).

### 5.5 Coordinated mitophagy and pexophagy during metastatic stress

Mitophagy and pexophagy remove different sources of organelle stress. BNIP3/NIX-dependent mitophagy eliminates damaged mitochondria, whereas mammalian pexophagy uses receptors and regulators including NBR1, PEX2, ATM-PEX5 and HIF-2α-dependent pathways (Deosaran et al., 2013; Sargent et al., 2016; Walter et al., 2014; Zhang et al., 2015). How the two fluxes are coordinated during metastasis is unknown, and steady-state organelle number cannot resolve it. Extensive pexophagy can reduce peroxide production but also removes catalase, fatty-acid-processing and ether-lipid capacity. Mitochondrial quality control can additionally clear mistargeted peroxisomal membrane proteins when peroxisome biogenesis fails (Segev-Nakar and Koren, 2026), illustrating reciprocal rather than independent stress management.

## 6 The mitochondria–peroxisome interface: structural and metabolic architecture

Mitochondria and peroxisomes are often described as partners in lipid oxidation and redox control. That description is useful, but it hides the variety of interactions involved. The organelles share terminal fission machinery, move along related cytoskeletal routes, exchange metabolites and oxidants, and participate in each other’s biogenesis and protein quality control. Evidence is strongest for biochemical coupling; physical contacts are now well supported in several systems, whereas direct causal links to cancer invasion remain comparatively sparse. The structural and pharmacological logic of this interface is summarised in Figure 2.

### 6.1 Physical and functional communication between mitochondria and peroxisomes

Systematic mapping in yeast identified peroxisome–mitochondria tethers and linked the contact to fatty-acid metabolism (Shai et al., 2018). Mammalian cells appear to use more than one architecture. ACBD2/ECI2 promotes close apposition in steroidogenic cells, and MFN1 or MFN2 can increase mitochondria–peroxisome clustering (Fan et al., 2016; Huo et al., 2022). These observations establish physical communication, but not a universal tether or a single function for every contact.

Recent work illustrates that functional heterogeneity. Fzo1-mediated contacts in yeast transfer peroxisomal citrate to mitochondria under defined metabolic conditions (Alsayyah et al., 2024), whereas mammalian ACBD5–PTPIP51 contacts can channel mitochondrial oxidants into peroxisomes for detoxification (DiGiovanni et al., 2025). A contact may therefore support metabolite exchange, redox buffering, organelle positioning or quality control depending on cell state. In metastatic models, the key experiment is still missing: perturb the contact while preserving organelle abundance and core enzyme expression, then test invasion or colonisation. Until that is done, contact-dependent effects on metastasis remain mechanistically plausible rather than established. The contact network is also not confined to the ER, mitochondria and peroxisomes: transferrin-containing endosomes make transient, functionally characterised contacts with mitochondria through which iron is delivered directly (Das et al., 2016; Hamdi et al., 2016), a route taken up in Section 9.3.

### 6.2 Shared division, distribution, and biogenesis

The two organelles use overlapping terminal fission machinery but distinct upstream membrane-remodelling systems. PEX11β elongates and constricts peroxisomal membranes before MFF/FIS1 and DRP1 complete scission (Itoyama et al., 2013; Koch and Brocard, 2012; Koch et al., 2005; Schrader et al., 2022). Mitochondrial DRP1 recruitment, by contrast, is integrated with mitochondrial adaptors, ER pre-constriction and actin-dependent membrane remodelling. Blocking DRP1 can therefore inhibit scission at both organelles. Targeting PEX11β would be predicted to bias the effect towards peroxisomes, but no cancer-selective PEX11β-directed pharmacological agent has yet established this selectivity.

Distribution and biogenesis provide further links. MIRO1 drives long-range peroxisome movement as well as mitochondrial transport (Castro et al., 2018), although coordinated movement during cancer-cell migration has not been demonstrated. Newly formed mammalian peroxisomes can arise from ER- and mitochondria-derived pre-peroxisomal vesicles (Sugiura et al., 2017), and mitochondria clear mistargeted peroxisomal membrane proteins when biogenesis fails (Segev-Nakar and Koren, 2026). Stress at one organelle can thus reshape the abundance and proteostatic burden of the other.

### 6.3 Coupled fatty-acid oxidation, omega oxidation, and cofactor exchange

The best-established metabolic partnership concerns fatty-acid chain length. Peroxisomes shorten very-long-chain and branched fatty acids that are inefficient mitochondrial substrates. Medium-chain acyl products, acyl-carnitines, acetyl units and propionyl units can then leave the peroxisome for further metabolism, including completion of oxidation in mitochondria (Violante et al., 2019). Because the peroxisomal pathway does not couple its first dehydrogenation step to ATP generation, it is better understood as preprocessing, overflow metabolism and detoxification than as a second mitochondrial power plant.

A parallel route begins in the endoplasmic reticulum. CYP4-family enzymes, including CYP4F11 for selected substrates, oxidise the terminal carbon of fatty acids and generate dicarboxylic products (Dhar et al., 2008). Long-chain dicarboxylic acids are then activated and shortened by peroxisomal β-oxidation rather than mitochondrial β-oxidation enzymes (Ferdinandusse et al., 2004). This ER-peroxisome pathway can expand when conventional mitochondrial fatty-acid oxidation is saturated or impaired, but its quantitative contribution in metastatic cells remains unknown. Dicarboxylates and chain-shortened products may nevertheless provide pharmacodynamic evidence that this pathway has been engaged.

Peroxisomes also contribute to acetyl-CoA and redox homeostasis. Stable-isotope tracing has shown that, when canonical cytosolic or mitochondrial acetyl-CoA production is constrained, peroxisomal oxidation of exogenous lipids can supply carbon for lipogenesis and histone acetylation (Kuna et al., 2023). At the same time, β-oxidation reduces NAD^+^ to NADH inside the peroxisome. Human cells maintain this ratio through lactate- and malate-based redox shuttles generated by readthrough isoforms of LDHB and MDH1 (Chornyi et al., 2023). These pathways couple peroxisomal flux to cytosolic biosynthesis, chromatin state and the redox environment in which mitochondria operate.

For metastasis, the open question is quantitative rather than merely anatomical: how much carbon and reducing power move through these routes at each stage of dissemination? Transcript levels of ACOX1 or ABCD3 cannot answer that. Isotope-resolved acyl-carnitine and dicarboxylate profiling, coupled to mitochondrial TCA labelling, is needed to distinguish substrate delivery from detoxification or signalling.

### 6.4 Why the interface matters in metastasis

The interface changes the conditions under which mitochondrial mechanometabolism is expressed. Peroxisomes influence which lipid substrates reach the mitochondrial system, how oxidants are buffered, which ether lipids populate invasive membranes, and how organelle quality control responds to stress. The conceptual advance is therefore not simply that two organelles are present, but that their coupling determines causal attribution and therapeutic window.

## 7 Cross-cutting amplifiers: organelle crosstalk, dynamics, and proteostasis

The four principal mitochondrial axes described in Sections 2–5 do not operate in isolation. Their effects are tuned by organelle-level processes that cut across spatial deployment, signalling, VDAC1-dependent exchange and hypoxic remodelling. This section considers mitochondria-ER contact sites, fission-fusion plasticity, mitochondrial proteostasis and the wider ER-mitochondria–peroxisome network as regulatory layers rather than additional independent axes.

### 7.1 Mitochondria–endoplasmic reticulum contact sites (MERCs)

MERCs are specialised membrane domains where the ER and OMM are held in close apposition (10–25 nm) by tethering protein complexes. Beyond the IP3R–GRP75–VDAC1 Ca^2+^ transfer complex described in Section 4.3, MERCs are sites of phospholipid transfer, autophagosome biogenesis, and mitochondrial fission (Awad and Abdul Karim, 2025; Shoshan-Barmatz et al., 2017a). The abundance and composition of MERCs are altered in several cancer models, but the direction and functional consequence are context dependent. In defined systems, increased contact density enhances mitochondrial Ca^2+^ uptake and oxidative metabolism, whereas altered lipid transfer can influence membrane organisation and vesicular traffic (Awad and Abdul Karim, 2025; Jin et al., 2023).

In addition to the IP3R–GRP75–VDAC1 complex, mitofusins contribute to ER-mitochondria organisation and the geometry of calcium and lipid-transfer microdomains (Han et al., 2021). MFN2 has been described as a tether in several systems, although genetic and ultrastructural studies also indicate context-dependent effects on contact distance and architecture. It is therefore safer to treat MFN2 as a regulator of contact organisation rather than as an invariant physical tether. Disruption of MERC components can impair mitochondrial Ca^2+^ uptake and bioenergetic adaptation, but the direction and magnitude of these effects vary across cancer models.

### 7.2 Mitochondrial fission–fusion dynamics and metastatic plasticity

The balance between mitochondrial fission and fusion determines mitochondrial morphology, function, and distribution. In the context of metastasis, this balance is dynamically regulated in response to metabolic and mechanical cues. DRP1-mediated fission is activated by multiple oncogenic kinases. ERK1/2 phosphorylates DRP1 at Ser616, promoting fission and mitochondrial fragmentation in RAS-mutant tumours (Kashatus et al., 2015; Serasinghe et al., 2015). CDK1 phosphorylates DRP1 at the same site during mitosis, ensuring mitochondrial segregation to daughter cells (Awad and Abdul Karim, 2025). In hepatocellular carcinoma, the receptor tyrosine kinase Met phosphorylates FIS1, promoting fission and metastasis (Yu et al., 2021). In ovarian cancer, IL-6 activates ERK1/2 to phosphorylate DRP1, enhancing mitochondrial fission and metastatic potential (Lee et al., 2024).

Beyond canonical kinase-driven regulation, mitochondrial fission is mechanosensitive, and in the opposite direction to the intuitive one. A soft extracellular matrix increases peri-mitochondrial F-actin through Spire1C and Arp2/3, driving DRP1- and MIEF1/2-dependent fission and raising mitochondrial ROS. The resulting oxidant load activates NRF2 and its cystine-uptake and glutathione programme, conferring resistance to ROS-dependent chemotherapy. *In vivo*, breast cancer cells disseminated to soft lung tissue displayed this phenotype, and combined inhibition of DRP1 and NRF2 restored cisplatin sensitivity (Romani et al., 2022) (Figure 3). MFN1-mediated fusion has context-dependent effects on metastasis. In HCC, MFN1 loss drives mitochondrial fragmentation, glucose metabolic reprogramming, and metastasis (Zhang et al., 2020). In lung adenocarcinoma, MFN1 mediates glucose-induced EMT (Liu X. et al., 2020b). Fusion-machinery components can nevertheless be required independently of network morphology. In an isogenic breast cancer series, metastatic cells displayed fragmented mitochondria with declining MFN1 and increasingly active DRP1, yet were selectively dependent on OPA1, which maintained tight cristae and high respiratory capacity; OPA1 deletion or the inhibitors MYLS22 and Opitor-0 reduced respiration, proliferation, migration and viability in metastatic cells while sparing their non-transformed counterparts (Diokmetzidou et al., 2025). OPA1-dependent cristae organisation, rather than a fused network as such, therefore appears to sustain respiratory competence in these cells. Together, these findings suggest that metastatic cells require dynamic switching between fission-dominant (for migration) and fusion-dominant (for colonisation) states, and that the ability to execute this switch is itself a determinant of metastatic fitness (Figure 1A).

**FIGURE 3:**
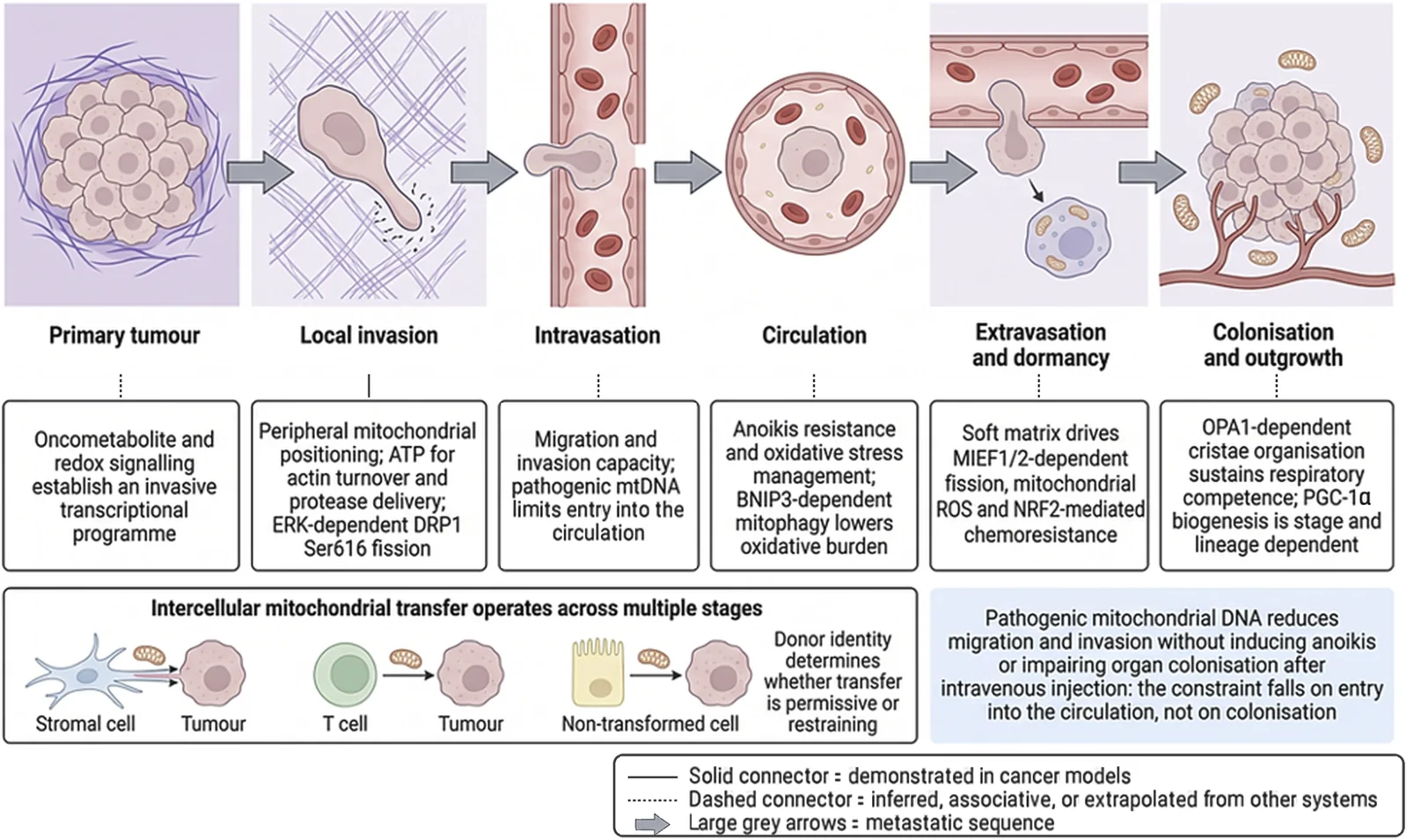
Stage-resolved mitochondrial requirements across the metastatic cascade. Local invasion has direct cancer-model support for peripheral mitochondrial positioning, ATP/protease delivery and ERK-DRP1 fission. Other stage assignments are presented more conservatively: oncometabolite/redox programmes in the primary tumour, BNIP3-dependent oxidative-stress management in circulation, soft-matrix MIEF1/2-ROS-NRF2 signalling at extravasation/dormancy, and OPA1/PGC-1α-linked respiratory competence during outgrowth. Pathogenic mtDNA reduces migration and invasion without inducing anoikis or preventing colonisation after intravenous injection, placing the principal constraint on entry into the circulation in that model. Intercellular mitochondrial transfer can operate across multiple stages and depends on donor identity. Solid connectors denote stage assignments demonstrated directly in cancer models; dashed connectors denote inferred, associative or extrapolated assignments; large grey arrows denote the metastatic sequence.

### 7.3 The mitochondrial unfolded protein response (UPR^mt^)

The mitochondrial unfolded protein response (UPR^mt^) is a retrograde programme activated when mitochondrial proteostasis is disrupted. Cancer cells exposed to hypoxia, respiratory-chain stress or chemotherapy can induce ATF5-dependent chaperones and proteases, including HSP60, mtHSP70 and LONP1 (Keerthiga et al., 2021). These responses may preserve respiratory competence and treatment tolerance. Direct evidence that a canonical UPR^mt^ programme is required for metastatic dissemination is still limited, however, and should be distinguished from the broader functions of mortalin/GRP75 at MERCs. Mortalin depletion sensitises drug-resistant BRAF-mutant melanoma cells through MEK/ERK- and ANT/CypD-dependent mechanisms (Wu et al., 2021), but this does not by itself establish that UPR^mt^ activation drives metastasis.

### 7.4 ER–mitochondria–peroxisome coordination

The ER provides a common organisational context for mitochondrial and peroxisomal metabolism. MERCs coordinate calcium transfer, phospholipid exchange, autophagosome formation and mitochondrial fission, while ER-peroxisome contacts support lipid trafficking and peroxisome growth. *De novo* peroxisome formation can involve both ER- and mitochondria-derived pre-peroxisomal vesicles (Sugiura et al., 2017), although the relative contribution of this pathway depends on cell type and the pre-existing peroxisome pool. Together, these observations support a three-organelle network in which the ER supplies membrane and lipid-processing capacity, peroxisomes initiate selected oxidative and ether-lipid pathways, and mitochondria convert substrates into ATP and signalling outputs. Endosome–mitochondria contacts add a fourth participant to this network: they are the route by which transferrin-derived iron reaches mitochondria without equilibrating with the cytosolic pool (Das et al., 2016; Hamdi et al., 2016), and they are discussed in Section 9.3, where iron availability is a stated determinant of ferroptotic susceptibility.

The relevance to invasion is plausible but not yet fully demonstrated. Migrating cells require coordinated membrane synthesis, focal-adhesion turnover, calcium signalling, and local energy production. An ER–mitochondria–peroxisome network could synchronise these demands, but most cancer studies quantify one contact pair at a time. Three-colour live imaging, split-fluorescent contact reporters, and correlative light-electron microscopy will be needed to distinguish stable tripartite junctions from rapid sequential encounters. Pharmacologically, targeting a shared ER-dependent trafficking or membrane-remodelling step may produce broader effects than targeting a mitochondria-specific channel such as VDAC1, again emphasising the need for organelle-resolved target validation.

## 8 Mitochondrial transfer in the tumour microenvironment

### 8.1 Mechanisms of intercellular mitochondrial transfer

The discovery that mitochondria can be transferred between cells via tunnelling nanotubes (TNTs), extracellular vesicles (EVs), and gap junctions has opened a new dimension in our understanding of metabolic communication in the tumour microenvironment (TME) (Guan et al., 2024; Saha et al., 2022; Zampieri et al., 2021). TNT-mediated transfer is the best-characterised mechanism: TNTs form between cancer cells and stromal cells or immune cells, and can transport intact mitochondria, mitochondrial fragments, and mtDNA over distances of tens to hundreds of micrometres (Guan et al., 2024; Pinto et al., 2021; Saha et al., 2022). While the evidence for intercellular mitochondrial transfer is now substantial, the field is still defining the quantitative contribution of transferred mitochondria to recipient cell metabolism, and the *in vivo* relevance beyond current experimental models remains an open question. The principal donor–recipient routes and their distinct consequences are summarised in Figure 3.

Mitochondrial transfer in the TME is bidirectional and context-specific. Stromal cells can donate functional mitochondria to cancer cells, increasing respiration and treatment tolerance in experimental models: adipose-derived stromal cells transfer mitochondria to breast cancer cells through tunnelling nanotubes, raising OXPHOS and conferring multidrug resistance (Del Vecchio et al., 2024), and melanoma cells recruit bone-marrow stromal cells and stimulate PGC-1α-dependent mitochondrial biogenesis in them, with transfer occurring by direct contact (Kumar et al., 2022). The general mechanisms of intercellular mitochondrial transfer have been reviewed elsewhere (Chen et al., 2025; Iorio et al., 2024). Donor identity is not incidental to the outcome: transfer from non-transformed mammary epithelial cells redirects breast cancer cells towards a normal progenitor fate and restrains tumoroid growth (Bjerring et al., 2025). A 2023 single-cell deconvolution analysis inferred T-cell-to-cancer mitochondrial transfer across human tumours, associating it with increased cancer-cell cycle activity and worse clinical outcome (Zhang et al., 2023). These results support cancer cells as mitochondrial recipients, but they do not exclude transfer in the opposite direction.

### 8.2 Bidirectional transfer between cancer cells and lymphocytes

Cancer-to-lymphocyte transfer represents a distinct immune-evasion mechanism. Analysis of clinical specimens and matched experimental models showed that cancer-cell mitochondria carrying mtDNA mutations can enter tumour-infiltrating T cells through direct-contact structures and small extracellular vesicles (Ikeda et al., 2025). The transferred mitochondria co-transported USP30, resisted mitophagy, and progressively replaced endogenous T-cell mitochondria. T cells that acquired the mutant mitochondrial genotype developed respiratory abnormalities, senescence, impaired effector and memory functions, and reduced responsiveness to PD-1 blockade (Ikeda et al., 2025). This mechanism should be distinguished from the T-cell-to-cancer transfer described by Zhang and colleagues (Zhang et al., 2023); both directions occur, but they benefit the tumour through different routes.

In glioblastoma, tunnelling-nanotube contacts mediate mitochondrial acquisition by tumour cells from infiltrating T cells, with associated effects on T-cell viability and LAG-3 expression, and the gap-junction inhibitor carbenoxolone reduces this transfer (Venkataratnam et al., 2025); nanotube-mediated transfer also occurs between glioblastoma stem cells and tumour organoids (Pinto et al., 2021). Frequency and functional contribution nevertheless remain model-dependent. TNT formation involves cytoskeletal regulators, including CDC42 and ROCK, but commonly used inhibitors such as cytochalasins and latrunculin are broad actin perturbants rather than transfer-selective drugs. Direction-specific reporters, donor-recipient genotyping and orthogonal transfer assays are required before a therapeutic effect is attributed specifically to mitochondrial exchange.

PGC-1α-driven mitochondrial biogenesis in stromal cells increases the supply of functional mitochondria available for transfer to cancer cells, enhancing cancer cell OXPHOS and resistance to immune killing (Kumar et al., 2022). Adipose-derived stem cells (ADSCs) transfer mitochondria to breast cancer cells via TNTs, driving multi-drug resistance through enhanced OXPHOS and reduced apoptotic sensitivity (Del Vecchio et al., 2024).

### 8.3 mtDNA transfer and metastatic enhancement

Beyond intact mitochondria, mtDNA can be transferred between cells via EVs, and this transfer can confer metastatic properties on recipient cells. Extracellular vesicles from highly metastatic tumour cells carry mtDNA with pathogenic mutations that, when transferred to low-metastatic cells, enhance their invasive and metastatic capacity in the systems studied (Takenaga et al., 2021). This horizontal transfer of oncogenic mtDNA represents a potential non-cell-autonomous mechanism of metastatic evolution that may contribute to the phenotypic heterogeneity of metastatic populations, though the frequency and clinical significance of this process remain to be established (Takenaga et al., 2021).

The effect of a transferred mitochondrion will also depend on the recipient cell. Peroxisomal fatty-acid processing, catalase reserve and ether-lipid remodelling can influence whether the acquired organelle improves OXPHOS or adds to oxidative stress. There is no comparable evidence for systematic intercellular transfer of peroxisomes in tumours; the more cautious interpretation is that the resident peroxisomal compartment conditions the fate of acquired mitochondria.

## 9 Peroxisomal control of mitochondrial metastatic fitness

Peroxisomal activity is not intrinsically pro- or anti-metastatic. The same organelle can broaden substrate use, reshape signalling lipids and support stress tolerance, yet also increase H_2_O_2_ or load membranes with ferroptosis-sensitive lipids. The outcome depends on flux, lipid species, antioxidant reserve, cell type and stage of dissemination.

### 9.1 Peroxisomal lipid metabolism and mitochondrial substrate flexibility

Peroxisomal β-oxidation can protect cells from poorly metabolised very-long-chain fatty acids and generate shorter products that mitochondria can use. This may be particularly valuable during colonisation of lipid-rich organs or when mitochondrial fatty-acid import is constrained. The cancer evidence for this route is directionally inconsistent and does not at present support a simple pro-metastatic reading. In colorectal cancer, SLC9A5 suppresses ACOX1-mediated peroxisomal β-oxidation, and it is loss of that oxidative programme, rather than its activation, that accompanies proliferation, migration and invasion (Zheng and Wang, 2023). In hepatocellular carcinoma, ACOX1 activation following SIRT5 loss is tumourigenic (Chen et al., 2018). We are not aware of a study that has tested peroxisomal β-oxidative flux against a metastatic endpoint in a single model with both gain and loss of function, and that is the experiment this argument requires.

That phenotype should not be equated with direct ATP production. Peroxisomal oxidation may help through detoxification, substrate provision, redox signalling or lipid remodelling. The relative contribution of each route is likely to change between glycolytic invasion and OXPHOS-dependent colonisation, and it must be resolved with flux measurements rather than organelle counts or transcript abundance.

### 9.2 Ether lipids, plasmalogens, and membrane-dependent cell behaviour

Ether-lipid synthesis begins in peroxisomes, where FAR1 supplies fatty alcohol and GNPAT and AGPS initiate the pathway; later steps occur in the ER. The products influence membrane packing, curvature, receptor organisation and the availability of signalling lipids. AGPS upregulation promotes aggressive cancer phenotypes, and chemical inhibition can reverse selected ether-lipid changes and suppress epithelial–mesenchymal features (Benjamin et al., 2013; Stazi et al., 2019). These observations make peroxisomal lipid synthesis a credible contributor to protrusion, vesicle traffic and invadopodial membrane turnover, although direct mechanical measurements remain limited.

Plasmalogens should not be treated as a single functional pool. Headgroup, saturation and the sn-2 acyl chain determine whether a species stabilises a membrane, releases arachidonate or becomes a peroxidation substrate. This species-level view is also important in immune cells. Loss of peroxisome-derived ether phospholipids compromises neutrophil membrane composition and viability (Lodhi et al., 2015), while ethanolamine-plasmalogen deficiency reduces macrophage phagocytosis and can be rescued by lysoplasmalogen supplementation (Rubio et al., 2018). A tumour-directed ether-lipid therapy could therefore alter antitumour immunity as well as cancer-cell membranes.

### 9.3 Ferroptosis at the mitochondrial–peroxisomal interface

The same pathway that supports membrane organisation can create a therapeutic liability. Peroxisome-dependent synthesis of polyunsaturated ether phospholipids is a determinant of ferroptosis susceptibility (Zou et al., 2020). Basal ether-lipid production may favour tumour fitness, yet those lipids become oxidisable substrates when GPX4 or system x_c_^−^ defences fail. Peroxisome abundance alone is therefore a poor predictor; the relevant variables are PUFA-ether-lipid composition, glutathione and GPX4 reserve, FSP1-CoQ protection, and iron availability (Doll et al., 2019).

Mitochondria shape a different part of this response through ROS, iron–sulfur metabolism and redox-cofactor supply. BNIP3 restrains NCOA4-dependent ferritinophagy and thereby limits the labile iron pool in melanoma (Vara-Pérez et al., 2021), whereas peroxisomal lipid synthesis increases the membrane substrate available for peroxidation. Both processes can operate in the same cell. This helps explain why erastin should be viewed primarily as a system x_c_^−^ inhibitor rather than a VDAC drug, and why rational combinations require lipidomic and iron-state stratification.

Oxidative stress is a major constraint on blood-borne melanoma dissemination: circulating and visceral metastatic cells experience oxidative stress, and antioxidant treatment can increase distant metastasis (Piskounova et al., 2015). Melanoma cells travelling through blood are also more GPX4-dependent than cells travelling through lymph, whose lipid and redox environment protects against ferroptosis (Ubellacker et al., 2020). These studies identify circulation as a particularly relevant setting in which to test peroxisome-derived PUFA ether lipids, but they do not directly compare ether-lipid dependence across all metastatic stages.

The therapeutic direction is therefore not one-dimensional. Peroxisome-derived PUFA ether phospholipids provide oxidisable substrates for ferroptosis; depleting them could protect disseminating cells from ferroptosis even if the same intervention suppresses pro-invasive membrane signalling in another context. Conversely, preserving or increasing oxidisable ether lipids may increase sensitivity to GPX4 inhibition. Stage-resolved experiments are needed to determine the net effects on invasion, survival in transit and colonisation; the current evidence does not justify ranking one stage as universally most dependent on the ether-lipid pool (Zou et al., 2020).

Iron availability adds a second axis. NCOA4-dependent ferritinophagy releases stored iron, while transferrin-containing endosomes can deliver iron directly to mitochondria through transient contacts demonstrated in erythroid and epithelial cells (Das et al., 2016; Hamdi et al., 2016). These endosome-mitochondria contacts therefore complement the ER-mitochondria-peroxisome network discussed in Sections 6.1 and 7.4.

The phenotypic consequences are lineage- and endpoint-dependent. BNIP3 loss raises labile iron and reduces melanoma tumour growth through PHD2-dependent HIF-1α degradation (Vara-Pérez et al., 2021), whereas DMT1 silencing in triple-negative breast cancer raises labile iron, activates PINK1/Parkin-dependent mitophagy and increases lung metastatic outgrowth, with DMT1-dependent endosome-mitochondria iron transfer differing between MDA-MB-231 and T47D cells (Barra et al., 2024). Because these studies use different tumour lineages and endpoints, they do not constitute a matched test of metastatic direction; they instead show that a larger labile iron pool can accompany distinct tumour phenotypes depending on the route and cellular context. Iron-state stratification should therefore specify both level and source.

### 9.4 PPAR–PGC-1α regulation of dual-organelle adaptation

PGC-1α is best known as a mitochondrial biogenesis coactivator, but its partnership with PPARs coordinates a wider oxidative programme. PPARα promotes mitochondrial and peroxisomal fatty-acid metabolism in responsive tissues; PPARδ and PPARγ also alter lipid uptake, differentiation, inflammatory signalling and immune-cell metabolism. The result depends on receptor isoform, tumour lineage and cellular compartment rather than on a generic increase in ‘peroxisome activity’.

Preclinical studies illustrate the ambiguity. Fenofibrate suppressed angiogenesis and tumour growth in several mouse models, and PPARα activation reduced melanoma-cell metastatic potential through Akt downregulation (Grabacka et al., 2006; Panigrahy et al., 2008). Yet fibrates can exert receptor-independent mitochondrial effects, and PPAR signalling may support fatty-acid use in other tumours. Rodents also display stronger hepatic peroxisome-proliferative responses to PPARα agonists than humans, limiting direct translational extrapolation. Genetic receptor dependence, direct target engagement and species-appropriate models are therefore essential before a cancer phenotype is attributed to PPAR activation.

The stage-specific PGC-1α behaviour described in breast cancer and melanoma extends to the interface. A PGC-1α-high state may favour OXPHOS and colonisation while increasing antioxidant and lipid-handling capacity; a PGC-1α-low state may favour glycolytic invasion but create reliance on alternative peroxisomal flux. Mapping the induced mitochondrial, peroxisomal and immune-cell programmes will be more informative than classifying the pathway as uniformly pro- or anti-metastatic.

### 9.5 MAVS, cGAS–STING and immune-cell immunometabolism

MAVS illustrates shared organelle localisation without identical kinetics. Early work separated rapid peroxisomal signalling from a delayed mitochondrial interferon response, while later studies showed that either pool can generate type I/III interferons in some systems (Bender et al., 2015; Dixit et al., 2010). An inducible study reported faster peroxisomal MAVS oligomerisation with similar downstream endpoints (Ramos et al., 2026); this kinetic model is recent and awaits independent replication.

In the tumour microenvironment, organelle signalling intersects with immune-cell metabolism. Peroxisome-derived ether lipids support neutrophil viability and macrophage phagocytosis, and peroxisomal metabolism contributes to macrophage engulfment and oxidative-burst resolution (Di Cara et al., 2017; Lodhi et al., 2015; Rubio et al., 2018). Mitochondrial fitness is likewise central to lymphocyte function. A perturbation that benefits tumour-cell killing can therefore have a different consequence in stromal or immune cells.

Duration and cellular context also matter for innate immune signalling. Persistent cGAS-STING activity can support non-canonical NF-κB-dependent invasion in chromosomally unstable tumours, prolonged interferon exposure can establish resistance programmes to checkpoint blockade, and constitutive STING signalling can stress T cells (Bakhoum et al., 2018; Benci et al., 2016; Wu et al., 2019). These observations support temporal and cell-type-resolved measurements rather than assuming that greater pathway activation is uniformly beneficial.

Mitochondrial damage can feed mtDNA-cGAS-STING and mitochondrial MAVS, whereas peroxisomal MAVS provides a distinct signalling platform; RIG-I-MAVS and cGAS-STING should not be conflated simply because both converge on interferon and NF-κB. The faster peroxisomal MAVS kinetics reported by Ramos et al. do not establish shorter or less exhausting tumour signalling. Similarly, the modest activity of the STING agonist MIW815 plus spartalizumab in a phase Ib study motivates investigation of delivery, cellular targeting, scheduling and tumour context, but does not identify selectivity rather than potency as the limiting factor (Meric-Bernstam et al., 2023).

Accordingly, organelle-restricted perturbation, temporal cytokine profiling and immune-competent metastatic models are needed before MAVS or STING can be assigned an organelle-specific anti-metastatic role.

## 10 Controversies and unresolved questions

### 10.1 Where is the pro-metastatic ROS window?

The idea that moderate ROS promote invasion whereas high ROS kill the cell remains useful, but ‘ROS level’ is not a complete variable. Species, duration, location and antioxidant reserve determine the response. Mitochondrial Complex I or III, peroxisomal ACOX1 and contact-mediated oxidant transfer can all influence the same cytosolic targets (Chen et al., 2018; DiGiovanni et al., 2025; Liu et al., 2023; Wang et al., 2023). A total-cell dye or a change in MitoSOX fluorescence cannot by itself identify the initiating compartment. The unresolved question is therefore where the signal begins, where it is buffered and which target experiences it.

### 10.2 BNIP3: Death effector or metastatic survival factor?

BNIP3 was first characterised as a pro-death BCL-2-family protein and is epigenetically silenced in several cancers. In hypoxic or nutrient-poor settings, however, its mitophagy-receptor activity removes damaged mitochondria, lowers oxygen demand and can protect against ferroptosis (Cao et al., 2023; Gao et al., 2020; Li et al., 2022; Li et al., 2026; Marinkovic et al., 2021; Mauro-Lizcano et al., 2024; Panigrahi et al., 2020; Poole and Macleod, 2021; Vara-Pérez et al., 2021; Wang et al., 2024). These functions are not mutually exclusive. Dimerisation, post-translational regulation, autophagy competence and nutrient state determine whether BNIP3 pushes a cell towards death or preserves a mitochondrial pool suited to invasion and treatment resistance.

### 10.3 mtDNA mutations: Drivers, passengers, or barriers?

Horizontal transfer of pathogenic mtDNA can increase metastatic traits in recipient cells, yet severe mtDNA mutations that impair respiratory-complex assembly can suppress melanoma metastasis (Shelton et al., 2024; Takenaga et al., 2021). The constraint in that model fell on intravasation rather than on colonisation: pathogenic mtDNA reduced migration and invasion without inducing anoikis or impairing organ colonisation after intravenous injection (Shelton et al., 2024). Metastatic selection may therefore act on mitochondrial function in a step-specific manner (Figure 3), tolerating dysfunction that would be prohibitive earlier in the cascade. Mutation identity, heteroplasmy and the metabolic demands of the target organ are likely to determine the outcome.

### 10.4 VDAC1 targeting: dependence versus normal-tissue function

VDAC1 is attractive because tumour cells often depend heavily on its metabolite exchange and protein interactions, but the channel is also essential in normal mitochondrial physiology (Camara et al., 2017; Magri et al., 2018). The therapeutic problem is to disrupt cancer-enriched states—such as VDAC1–HK-II association, abnormal expression or selected oligomeric assemblies—without broadly blocking mitochondrial exchange. Delivery, structural selectivity and metabolic bypass are likely to determine whether the preclinical window survives *in vivo* (Arif et al., 2024; Huang et al., 2025; Reina and De Pinto, 2017; Shoshan-Barmatz et al., 2017b; Shteinfer-Kuzmine et al., 2024).

### 10.5 Shared machinery and opposing lipid outcomes

DRP1-directed pharmacology illustrates the danger of organelle labels (Figure 4). Mdivi-1 inhibits Complex I and alters ROS independently of DRP1 (Bordt et al., 2017); P110 more specifically disrupts DRP1–FIS1, but FIS1 also participates in peroxisome division (Koch et al., 2005; Qi et al., 2013; Schrader et al., 2022). An anti-migratory phenotype may therefore arise from mitochondrial fusion, altered peroxisomal division, respiratory inhibition or a combination. Each mechanism predicts a different biomarker and toxicity profile.

**FIGURE 4:**
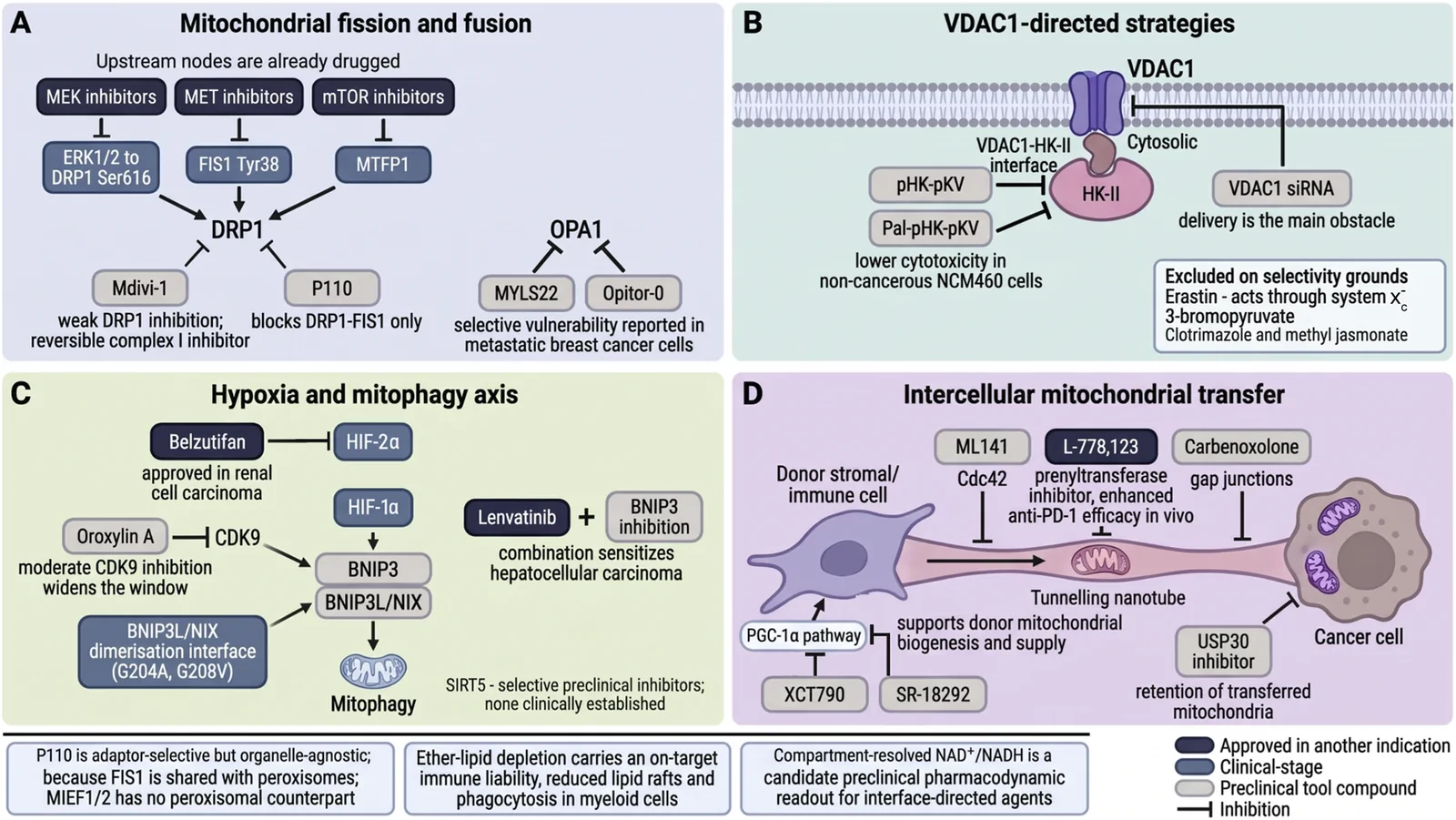
Pharmacological entry points and the selectivity problem. Compound boxes are shaded by development stage. **(A)** Mdivi-1 is a weak DRP1 inhibitor and reversible Complex I inhibitor; P110 disrupts DRP1-FIS1, while clinically used kinase classes act upstream of ERK1/2-DRP1, FIS1-Tyr38 and MTFP1. MYLS22 and Opitor-0 probe OPA1 dependence reported in metastatic breast-cancer cells. **(B)** pHK-pKV/Pal-pHK-pKV target the VDAC1-HK-II interface, whereas VDAC1 siRNA reduces VDAC1 expression; erastin is excluded as a VDAC-selective agent. **(C)** Belzutifan targets HIF-2α; BNIP3L/NIX dimerisation and CDK9-dependent BNIP3 expression provide downstream experimental nodes, while selective SIRT5 inhibitors remain preclinical and BNIP3 inhibition can sensitise hepatocellular carcinoma to lenvatinib. **(D)** ML141 targets Cdc42, L-778,123 and carbenoxolone can reduce transfer-related processes, USP30 inhibition acts on persistence of transferred mitochondria, and donor-side PGC-1α pathway suppression provides a complementary strategy. The lower boxes emphasise adaptor versus organelle selectivity, myeloid ether-lipid liability and compartment-resolved NAD^+^/NADH as a candidate preclinical pharmacodynamic readout.

Peroxisomal lipid metabolism presents a parallel ambiguity. Ether-lipid synthesis can support motility and treatment persistence (Benjamin et al., 2013; Stazi et al., 2019), while ACOX1-derived H_2_O_2_ and PUFA-ether phospholipids can create oxidative or ferroptotic liabilities (Chen et al., 2018; Zou et al., 2020). Peroxisomal β-oxidation itself resists a single assignment: its suppression accompanies an aggressive phenotype in colorectal cancer (Zheng and Wang, 2023) while its activation is tumourigenic in liver (Chen et al., 2018). The relevant unit of analysis is a defined flux and lipid species at a defined metastatic stage—not peroxisome abundance in aggregate.

## 11 Therapeutic implications

### 11.1 Targeting mitochondrial dynamics

DRP1 suppression reduces migration and invasion in several preclinical cancer models (Chen et al., 2023; Chung et al., 2021; Tian et al., 2025), and DRP1 expression and Ser616 phosphorylation predict postoperative recurrence risk in stage I–IIIA lung adenocarcinoma (Chung et al., 2021). The pharmacological evidence must nevertheless be interpreted carefully. Mdivi-1 is a perturbation tool rather than a clinical lead: it inhibits recombinant DRP1 GTPase only weakly, reversibly inhibits Complex I, and alters respiration in cells lacking DRP1 entirely (Bordt et al., 2017). P110 disrupts the DRP1–FIS1 interaction specifically and spares DRP1 binding to its other adaptors (Qi et al., 2013); as discussed in Section 11.5.1, that selectivity is selectivity for the adaptor shared with peroxisomes. A more tractable route may lie upstream, because the kinases that activate DRP1 are already drugged: ERK1/2-dependent Ser616 phosphorylation is blocked by MEK inhibition (Kashatus et al., 2015; Serasinghe et al., 2015), CDK2 and AKT contribute in lung adenocarcinoma (Chung et al., 2021), Met phosphorylates FIS1 at Tyr38 to recruit DRP1 in hepatocellular carcinoma (Yu et al., 2021), and the p53–mTORC1–MTFP1 axis sets DRP1 phosphorylation and dissemination in a manner abolished by blocking mTORC1 (Phan et al., 2022). None of these agents is selective for mitochondrial fission, but each represents existing clinical chemical matter acting on a node for which no direct inhibitor has reached the clinic. Where fusion machinery is required, OPA1 offers a distinct vulnerability with chemistry attached: OPA1 deletion or the inhibitors MYLS22 and Opitor-0 reduce respiration, proliferation, migration and viability in metastatic breast cancer cells while sparing non-transformed counterparts (Diokmetzidou et al., 2025).

### 11.2 VDAC1-directed and VDAC1-associated strategies

VDAC1-directed peptides and siRNA provide a comparatively mitochondria-restricted pharmacological logic (Table 1). Peptides engineered from the VDAC1-binding N-terminus of HK-II—pHK-pKV and its palmitoylated self-assembling form Pal-pHK-pKV—localise to mitochondria and kill A549 lung carcinoma cells, and show substantially lower cytotoxicity in non-cancerous NCM460 cells (Liu et al., 2019); related amphiphile designs act in melanoma and cervical carcinoma models (Sun et al., 2025; Zhang et al., 2021). This cancer-versus-normal comparison is the most directly relevant therapeutic-window datum in the section. VDAC1-based peptides more broadly disrupt cancer-associated protein interactions and reduce tumour growth (Shteinfer-Kuzmine et al., 2024), while VDAC1 silencing produces broader metabolic reprogramming and reduces stemness and invasion, with delivery the principal obstacle (Arif et al., 2024). Two classes are deliberately excluded. Erastin appears in Table 1 only as a comparator: its dominant ferroptotic mechanism is system x_c_^−^ inhibition and glutathione depletion, and direct VDAC involvement remains debated (Huang et al., 2025; Reina and De Pinto, 2017). The historical HK-II-dissociating agents are omitted because their specificity and toxicity profiles do not meet the selectivity standard argued for here (Krasnov et al., 2013).

**TABLE 1:** Interface and VDAC1 targets: Interface mechanism, development stage, pharmacodynamic readouts and principal limitations.

Target/pathway	Representative intervention	Interface mechanism	Evidence stage	Pharmacodynamic readout	Main limitation
DRP1–MFF–FIS1	Mdivi-1 (tool); P110; conceptual adaptor-selective strategies	Shared mitochondrial and peroxisomal scission	Tool compound/preclinical	Dual-organelle morphology; respiration; peroxisome number	Complex I off-targets; peroxisomal effects unresolved
ACOX1–catalase/contacts	ACOX1 or catalase modulation; contact modulators (conceptual)	Peroxisomal FAO, H_2_O_2_ production and disposal of mitochondrial oxidants	Genetic/preclinical; no validated contact drug	Targeted H_2_O_2_ sensors; ACOX flux; contact frequency	Bidirectional redox effects; systemic lipid toxicity
AGPS–FAR1–GNPAT	AGPS inhibitors; ether-lipid combinations	Plasmalogen and PUFA-ether-phospholipid synthesis	Preclinical	Species-resolved ether lipidomics; membrane and EMT readouts	May reduce invasion and ferroptotic sensitivity simultaneously
GPX4/system x_c_^-^	Erastin-class agents; GPX4 inhibitors	Exploits oxidisable peroxisome-derived ether lipids	Preclinical	GPX4 dependence; oxidised ether lipids; labile iron	Normal-tissue toxicity; strong lipid-context dependence
PPARα–PGC-1α	Fenofibrate and other PPAR ligands	Coordinates mitochondrial biogenesis and peroxisomal FAO	Clinical in metabolic disease; preclinical cancer repurposing	Receptor target genes; OXPHOS; acyl-carnitines	Pleiotropy; receptor-independent and immune effects
BNIP3/NIX–NBR1–PEX2	Mitophagy/pexophagy and ubiquitin-pathway modulators	Dual-organelle quality control under hypoxia and stress	Genetic/preclinical	Paired flux reporters; organelle abundance and turnover	Broad autophagy and proteostasis effects
MAVS/cGAS–STING	RLR/MAVS or STING modulators	Organelle- and cell-type-specific innate immune signalling	Conceptual for organelle-specific metastasis therapy	Cytokine kinetics; tumour versus immune-cell activation	Chronic inflammation; no organelle-selective anticancer agent
VDAC1–HK-II interface (comparator)	VDAC1–HK-II-targeted peptide amphiphiles: pHK-pKV and Pal-pHK-pKV (Liu et al., 2019; Sun et al., 2025; Zhang et al., 2021)	Disrupts HK-II docking on the OMM and restores apoptotic sensitivity; predominantly mitochondria-restricted, with no peroxisomal counterpart	Preclinical (lung, melanoma and cervical carcinoma models)	HK-II displacement from mitochondria; apoptotic index; cancer-versus-normal cytotoxicity (lower in NCM460 cells)	Delivery and biodistribution; VDAC proteins essential in normal tissue
VDAC1 expression (comparator)	VDAC1 siRNA (Arif et al., 2024)	Lowers OMM metabolite exchange, producing broad metabolic and transcriptional reprogramming	Preclinical, multiple solid-tumour models	VDAC1 abundance; respiration; stemness and invasion markers	Delivery; possible isoform compensation by VDAC2/VDAC3
VDAC1 protein–protein interactions (comparator)	VDAC1-based peptides (Shteinfer-Kuzmine et al., 2024)	Disrupts cancer-enriched VDAC1 interactions rather than blocking the channel	Preclinical, multiple models	Interaction disruption; tumour growth; tumour-microenvironment composition	Selectivity for cancer-enriched states not established *in vivo*
system x_c_^−^ (excluded; listed for contrast)	Erastin (Huang et al., 2025; Reina and De Pinto, 2017)	Induces ferroptosis through system x_c_^−^ inhibition and glutathione depletion; direct VDAC involvement debated	Preclinical	Glutathione depletion; lipid peroxidation; GPX4 dependence	Not a VDAC-selective agent; retained only as a comparator for the selectivity argument
Biomarker platform	Targeted metabolomics; isotope tracing; spatial lipidomics	Classifies organelle engagement and treatment response	Enabling technology	Acyl-carnitines; dicarboxylates; ether lipids; oxidised PLs; NAD ratios	Standardisation, spatial resolution and prospective validation

Development stage is stated for every entry, and ‘organelle-selective’ is used only where the distinction has been measured rather than assumed. Section 11.5.1 sets out the six-assay panel—cellular target engagement, paired dual-organelle morphometry, organelle-resolved adaptor recruitment, genetic epistasis against FIS1 and MIEF1/MIEF2, biochemical peroxisomal flux, and compartment-targeted redox probes—by which an adaptor-selective agent can be distinguished from an organelle-selective one. No agent in the DRP1–MFF–FIS1 row has completed that panel. VDAC1-directed strategies are included as the mitochondria-restricted comparator against which interface-spanning targets should be judged, and as the case in which an organelle-restricted target logic is already available.

### 11.3 Targeting the HIF-1α–BNIP3 axis

The HIF-1α-BNIP3 pathway contains several intervention points. Belzutifan validates HIF pharmacology clinically but targets HIF-2α rather than HIF-1α (Choueiri et al., 2024). BNIP3L/NIX dimerisation depends on transmembrane residues including G204 and G208, while CDK9-dependent BNIP3 expression is addressable with oroxylin A (Li et al., 2022; 2026; Marinkovic et al., 2021; Yao et al., 2022). SIRT5 is mechanistically attractive but context dependent; selective inhibitors exist preclinically, although none is clinically established (Fiorentino et al., 2022; Liu et al., 2021; Zhang et al., 2025). BNIP3 inhibition can sensitise hepatocellular carcinoma to lenvatinib (Wang et al., 2024), underscoring the need for biomarker-defined context rather than pathway-wide assumptions.

### 11.4 Targeting mitochondrial transfer

Mitochondrial transfer is therapeutically attractive because it can support cancer-cell respiration or impair antitumour lymphocytes, but donor, recipient and direction must be specified. ML141 inhibits Cdc42 and can reduce nanotube-mediated transfer; L-778,123 partially blocks nanotube formation and enhanced anti-PD-1 efficacy in an immunocompetent breast-cancer model; carbenoxolone reduces mitochondrial acquisition in glioblastoma models (Saha et al., 2022; Venkataratnam et al., 2025). USP30 inhibition acts on persistence of transferred, mitophagy-resistant organelles rather than directly blocking nanotube formation (Ikeda et al., 2025). Donor-side PGC-1α pathway suppression with XCT790 or SR-18292 provides another strategy, although these compounds act through different mechanisms and should not be treated as equivalent direct PGC-1α inhibitors (Fontana et al., 2024; Kumar et al., 2022). Because transfer from non-transformed cells can also restrain tumour phenotypes, indiscriminate blockade may remove permissive and restraining inputs (Bjerring et al., 2025).

### 11.5 Pharmacological targeting of the mitochondria–peroxisome interface

The interface is best approached as a target-selection and validation problem rather than as a single drug class (Table 1). VDAC1 is largely mitochondria restricted; DRP1–MFF–FIS1, redox enzymes, lipid synthesis and selective autophagy span both organelles or converge on shared outputs. Development stage must therefore be stated explicitly, from clinically established PPAR ligands used in other indications to tool compounds, genetic targets and contact-site concepts without validated drugs.

#### 11.5.1 Shared fission and redox machinery

The selectivity problem can be stated precisely rather than as a general caution. FIS1 and MFF are shared between mitochondria and peroxisomes (Itoyama et al., 2013; Koch and Brocard, 2012; Koch et al., 2005; Schrader et al., 2022), whereas MiD49 and MiD51 (MIEF2/MIEF1) are not targeted to the peroxisomal surface and act specifically at mitochondria (Palmer et al., 2013). P110 blocks the DRP1–FIS1 interaction specifically while sparing DRP1’s other adaptors (Qi et al., 2013). P110 is therefore adaptor-selective but organelle-agnostic, because the adaptor it selects is the shared one; its peroxisomal consequences have not been measured and could occur; they should therefore be measured rather than assumed absent. The corollary identifies the organelle-selective alternative: a MIEF1/2-directed approach would act on mitochondrial fission without a direct peroxisomal counterpart, and that axis is neither hypothetical nor disease-irrelevant, since a soft extracellular matrix drives DRP1- and MIEF1/2-dependent fission, mitochondrial ROS and NRF2-mediated chemoresistance in disseminated breast cancer cells, where combined DRP1 and NRF2 inhibition restored cisplatin sensitivity (Romani et al., 2022). Redox interventions require the same discipline. ACOX1 inhibition changes both fatty-acid flux and H_2_O_2_; catalase changes buffering without blocking β-oxidation; contact-site manipulation alters disposal of mitochondrial oxidants (DiGiovanni et al., 2025). The desired direction depends on whether peroxisomes act as an oxidant source or a sink, and that sign is tissue-dependent (Section 3.4). Mitochondria-targeted catalase suppressed invasive progression in a mouse mammary-tumour model (Goh et al., 2011), although that evidence is genetic and the pharmacological equivalent has not been tested against metastatic endpoints.

Table 1 separates adaptor selectivity from organelle selectivity, which should be demonstrated rather than inferred from a target name. A practical validation panel should combine: (i) direct target engagement in intact cells, using CETSA where technically validated or an orthogonal binding/interaction assay; (ii) paired mitochondrial and peroxisomal morphometry; (iii) organelle-resolved DRP1 recruitment; (iv) genetic epistasis tailored to the claimed adaptor—for example, FIS1 loss for a FIS1-directed mechanism and MIEF1/2 perturbation for the mitochondrial-specific branch; (v) biochemical peroxisomal flux, because morphology and function can dissociate; and (vi) compartment-targeted redox probes (Ferdinandusse et al., 2004; Martinez Molina et al., 2013; Pak et al., 2020; Palmer et al., 2013; Violante et al., 2019). Lack of a statistically significant peroxisomal phenotype is not evidence of equivalence; predefined effect bounds and rescue controls are preferable. Until paired organelle-resolved measurements are available, organelle selectivity should be considered unestablished even when adaptor selectivity has been demonstrated.

#### 11.5.2 Ether-lipid and ferroptosis pharmacology

Ether-lipid synthesis is unusually tractable pharmacologically for an organelle-interface pathway. A structure-based series around AGPS yielded compound 2i, a 2,6-difluoro analogue of the first-in-class inhibitor with improved binding affinity, which lowers ether-lipid levels and migration in 231MFP cells and impairs EMT in PC-3 and MDA-MB-231 cells through E-cadherin, Snail and MMP2; the AGPS–inhibitor complex has been resolved crystallographically (Stazi et al., 2019). The effect will not be one-dimensional: reducing ether lipids may limit pro-invasive membrane signalling but also removes the PUFA-ether species that sensitise cells to ferroptosis, and it carries a measurable on-target liability in the immune compartment, since ethanolamine-plasmalogen depletion reduces the number and size of lipid rafts, increases membrane fluidity and impairs receptor-mediated signalling and phagocytosis in macrophages (Rubio et al., 2018), while peroxisomal lipid synthesis supports neutrophil viability (Lodhi et al., 2015). Raft integrity and receptor signalling in tumour-infiltrating myeloid cells are therefore candidate safety readouts rather than a generic caution. Ferroptosis combinations should be selected on species-resolved ether-lipid profiles, GPX4/system x_c_^−^ dependence, labile iron and mitochondrial redox state rather than peroxisome abundance.

#### 11.5.3 PPAR signalling, quality control, and innate immunity

PPARα agonists provide clinical precedent: fenofibrate reduces melanoma metastatic potential through Akt downregulation, with constitutively active Akt conferring resistance and Akt siRNA phenocopying the drug (Grabacka et al., 2006), and suppresses tumour growth through angiogenesis inhibition (Panigrahy et al., 2008). Receptor dependence should nonetheless be measured directly. Organelle-turnover pharmacology is further advanced than the phrase modulation of mitophagy or pexophagy conveys: the neddylation inhibitor MLN4924 (pevonedistat) induces pexophagy through HIF1α-dependent NIX upregulation, while the selective USP30 inhibitor CMPD-39 induces pexophagy through a distinct NBR1-dependent route (Barone et al., 2023). A HIF1α–NIX pexophagy axis with a defined chemical probe attached sits directly on the HIF-1α–BNIP3/NIX axis developed in Section 5.3. Independently, peroxisome abundance is reduced in VHL-deficient clear cell renal carcinoma with high HIF-2α (Walter et al., 2014), predicting that HIF-2α inhibition should raise peroxisome abundance—a testable, drug-linked interface prediction using belzutifan (Choueiri et al., 2024). MAVS- and STING-directed approaches add cell-type dependence: a signal that is immunogenic in a dendritic cell may be chronically inflammatory in a tumour cell.

#### 11.5.4 Metabolomic and lipidomic pharmacodynamic readouts

A practical interface programme needs biomarkers that report flux rather than expression alone. Candidate readouts include acyl-carnitine and dicarboxylate ratios, species-resolved ether lipids, oxidised PUFA-ether lipids, isotope incorporation into mitochondrial TCA intermediates or acetylated histones, and compartment-resolved redox measurements. Genetically encoded sensors establish that free NAD^+^ concentrations differ among subcellular compartments, but NAD^+^ concentration is not itself an NAD^+^/NADH ratio and such sensors currently require experimental genetic delivery (Cambronne et al., 2016). ^31^P MRS can quantify tissue NAD metabolites and redox state *in vivo*, demonstrated in human brain, but this does not yet constitute a validated organelle-resolved pharmacodynamic assay for solid tumours (Lu et al., 2016). *Ex vivo* tumour measurements additionally require controlled collection-to-quench intervals because ischaemia can alter metabolite profiles (Ricci et al., 2024). We therefore treat compartment-resolved NAD^+^/NADH as a candidate preclinical mechanistic readout; tissue-level NAD measurements may provide complementary clinical information, but their relationship to organelle-specific drug effects requires prospective validation.

#### 11.5.5 Minimum target-validation framework

A candidate interface therapy should satisfy seven tests: direct target engagement; simultaneous measurement of mitochondrial and peroxisomal morphology; organelle-resolved redox analysis; fatty-acid and ether-lipid flux measurements; genetic rescue that separates mitochondrial, peroxisomal and non-organelle effects; testing in relevant stromal and immune cells; and metastatic end points that include invasion, survival in transit and colonisation. This framework is deliberately demanding. Without it, organelle crosstalk remains a descriptive explanation rather than a drug-discovery mechanism. It is easier to accept with a concrete instance. Met phosphorylates FIS1 at Tyr38, and Fis1-pY38 recruits DRP1 to drive fission and hepatocellular carcinoma metastasis (Yu et al., 2021); FIS1 is also the shared mitochondrial–peroxisomal fission adaptor. Whether Met-dependent FIS1-Tyr38 phosphorylation also alters peroxisome division is therefore a single-phosphosite, two-organelle experiment, testable with existing reagents and available MET inhibitors, and diagnostic of whether the interface framing predicts anything a mitochondria-only framing does not. We propose it as the minimal decisive experiment for this hypothesis.

## 12 Discussion and outlook

Mitochondria support metastasis through spatial deployment, redox and metabolite signalling, VDAC1-dependent exchange and HIF-1α-BNIP3-dependent remodelling. Fission-fusion plasticity, contact sites, proteostasis and intercellular mitochondrial transfer tune these functions across the metastatic cascade.

Peroxisomes modify rather than replace this framework. Fatty-acid shortening, dicarboxylate processing, redox buffering, ether-lipid synthesis, pexophagy and MAVS signalling alter mitochondrial substrate use, oxidative state and therapeutic interpretation (Chornyi et al., 2023; Ferdinandusse et al., 2004; Kuna et al., 2023). The interface is therefore most useful where it changes causal attribution or drug selectivity.

The evidence boundary remains important. Shared fission machinery, metabolic coupling and peroxisome-derived ether lipids are well established, but a direct causal role for mitochondria-peroxisome contacts in invasion, transit or colonisation has not been demonstrated. Oxidant transfer at ACBD5-PTPIP51 contacts was characterised without testing metastatic endpoints, and several tether architectures derive from yeast or non-malignant mammalian systems (DiGiovanni et al., 2025; Fan et al., 2016; Huo et al., 2022; Shai et al., 2018). These gaps define the next experiments: contact-specific perturbation without organelle loss, dual-organelle live imaging in mechanically defined matrices and paired mitophagy-pexophagy flux measurements in immune-competent models.

Pharmacologically, VDAC1-directed strategies can be evaluated primarily as mitochondrial interventions, whereas DRP1/FIS1 inhibition, redox modulation, PPAR signalling and autophagy manipulation require paired peroxisomal measurements. Ether-lipid interventions may suppress invasive signalling while reducing ferroptotic sensitivity, and innate-immune interventions require cell-type and duration-resolved assessment. Therapeutic selectivity therefore depends on demonstrating which organelle and cell population is engaged.

Mechanism should be connected to measurable flux: organelle-targeted redox sensors can resolve source-to-target oxidant transfer; isotope tracing can separate peroxisomal chain shortening from mitochondrial completion of oxidation; spatial lipidomics can map ether lipids and oxidised phospholipids; and single-cell or spatial profiling can identify tumour, stromal and immune states. Built into target validation, these measurements turn the interface from a descriptive network into a testable framework.

Several translational opportunities can be pursued with existing agents, but they should be framed as tests rather than established clinical strategies. MEK, MET and mTOR inhibitors provide pharmacological access to upstream fission control and could be paired with dual-organelle pharmacodynamic sampling. Belzutifan offers a setting in which the predicted relationship between HIF-2α inhibition and peroxisome abundance can be tested in on-treatment material (Choueiri et al., 2024; Walter et al., 2014). Clinical STING studies motivate better delivery, scheduling and cell-type-resolved readouts rather than stronger global activation alone (Meric-Bernstam et al., 2023). Ether-lipid-directed approaches should monitor myeloid function because plasmalogen depletion impairs macrophage phagocytosis and neutrophil viability (Lodhi et al., 2015; Rubio et al., 2018). These examples define a practical route from organelle biology to falsifiable clinical pharmacology.
